# Drug-Resistant Pandemic (H1N1) 2009, South Korea^1^

**DOI:** 10.3201/eid1704.101467

**Published:** 2011-04

**Authors:** Soo Youn Shin, Chun Kang, Jin Gwack, Joon Hyung Kim, Hyun Su Kim, Young A Kang, Ha Gyung Lee, Jin Seok Kim, Jong-Koo Lee, Sung-Han Kim

**Affiliations:** Author affiliations: Korea Centers for Disease Control and Prevention, Seoul, South Korea (S.Y. Shin, C. Kang, J. Gwack, J.H. Kim, H.S. Kim, Y.A. Kang, H.G. Lee, J.S. Kim, J.-K. Lee);; University of Ulsan College of Medicine, Seoul (S.-H. Kim)

**Keywords:** Pandemic (H1N1) 2009 virus, influenza, viruses, oseltamivir, antimicrobial resistance, antiviral drugs, South Korea, expedited, dispatch

## Abstract

Eleven patients with drug-resistant pandemic (H1N1) 2009 were identified in South Korea during May 2009–January 2010. Virus isolates from all patients had the H275Y mutation in the neuraminidase gene. One isolate had the I117M mutation. Of the 11 patients, 6 were <59 months of age, and 5 had underlying immunosuppressive conditions.

The Korea Centers for Disease Control and Prevention asked clinicians to report all patients with suspected cases of drug-resistant pandemic (H1N1) 2009 when these patients showed treatment failure for oseltamivir or had unusually prolonged viral shedding (defined as >5 days after the onset of symptoms) (*1*). We report nationwide surveillance data on the epidemiologic and clinical characteristics of patients infected with pandemic (H1N1) 2009 in South Korea.

## The Study

From the first reported infected patient in May 2009 through January 2010, a total of 740,835 patients in South Korea were reported as having pandemic (H1N1) 2009 virus infection. A total of 225 patients (0.03%) died of disease related to pandemic (H1N1) 2009. During this period, physicians in local clinics and tertiary hospitals sent specimens from 67 patients who were suspected of having drug-resistant pandemic (H1N1) 2009 to the Korea Centers for Disease Control; 11 patients (16%) had drug-resistant virus (Figure). After confirmation of drug resistance, Epidemic Intelligence Service officers obtained clinical and epidemiologic data by medical record review and interviews with household contacts and attending physicians for all patients.

**Figure:**
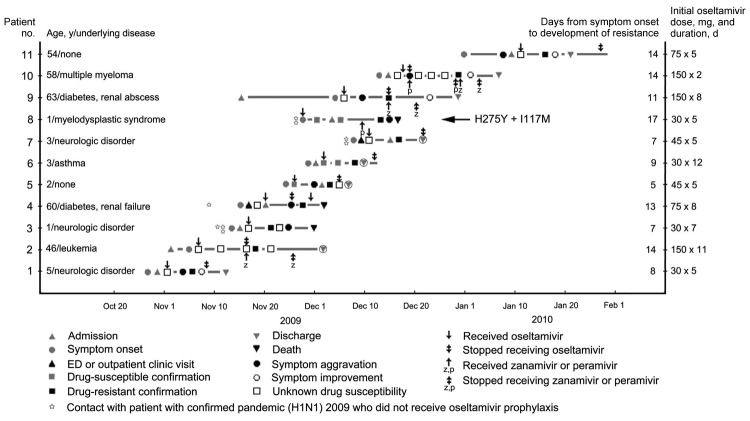
Clinical course and outcome of 11 patients with oseltamivir-resistant pandemic (H1N1) 2009, South Korea. Symptom aggravation was defined as influenza-related symptoms that worsened regardless of new infiltrations seen by chest radiography. Symptom improvement was defined as influenza-related symptoms (nasal stiffness, sore throat, cough, myalgia, fatigue, headache, and fever) that were absent or mild. All doses of oseltamivir were given 2×/d. ED, emergency department.

To investigate whether virus contained genetic markers associated with resistance to antiviral drugs, a conventional genotyping assay (sequencing) was performed, and neuraminidase (NA) and matrix 2 genes were sequenced. All 11 patients had virus with a histidine-to-tyrosine mutation at residue 275 of the NA protein (H275Y); 1 patient also had virus with an I117M mutation (Figure). Detailed molecular epidemiologic data and genetic characteristics of the isolates are described elsewhere (*2*).

Of the 11 patients, 6 were <59 months of age and 5 had underlying immunosuppressive conditions; only 1 patient was immunocompetent and >59 months of age (Table). Three patients from whom samples before and after treatment with oseltamivir were available showed evidence of having acquired the H275Y mutation during oseltamivir therapy. None of the 11 patients received oseltamivir chemoprophylaxis.

**Table:** Characteristics of 11 patients with oseltamivir-resistant pandemic (H1N1) 2009, South Korea*

Characteristic	Value
Median age, y (range)	5 (1–63)
Male sex	6
No. at high risk for influenza-related complications	
<59 mo of age	6
Chronic medical disorders	9
Neurologic	3
Hematologic	3
Metabolic, including diabetes†	2
Pulmonary, including asthma	1
Renal†	1
Cardiovascular, excluding hypertension	0
Hepatic disorder	0
Immunocompromised‡	5
Median days from symptom onset to viral isolation (range)	2 (0–9)
Median days from symptom onset to development of resistance	8
No. hospitalized	11
Hospitalization duration, d, median (range)	15 (6–53)
Symptom duration, d, median (range)	17 (6–22)
No. with respiratory illness related to influenza	11
Viral pneumonia	6
Secondary bacterial pneumonia	2
Secondary bacterial pneumonia and acute respiratory distress syndrome	1
Reasons for requesting drug-resistance testing	
Treatment failure	9
Prolonged viral shedding	2
No. with co-infections§	3
Outcome	
Cured	8
Died¶	3

*Specimens were obtained from oropharyngeal (n = 7) and nasopharyngeal (n = 2) swabs, nasopharyngeal washings (n = 1), and brochoalveolar lavage fluid (n = 1). †One patient had diabetes and chronic renal failure. ‡Patients who had underlying diseases such as HIV infection, malignancy, liver cirrhosis, or chronic renal failure, or patients receiving immunosuppressive treatment. §Carbapenem-resistant *Pseudomonas aeruginosa* (patient 3), carbapenem-resistant *Acinetobacter* *baumanii* (patient 4), and penicillin-susceptible *Streptococcus pneumonia* (patient 10). ¶Of the 3 patients, 1 was infected with carbapenem-resistant *P*. *aeruginosa* (patient 3) and 1 was infected with carbapenem-resistant *A*. *baumanii* (patient 4).

We also tested 100 persons who had contact with the 11 patients for possible transmission of drug resistance. Eight of 100 were confirmed as having been infected with pandemic (H1N1) 2009 virus before the 11 patients were infected. Influenza-like illnesses developed in the 11 patients a median of 2 days (range 1–7 days) after the 8 contact persons were confirmed as having pandemic (H1N1) 2009. Five of the 8 contact patients were children; none had an immunosuppressive condition or were given oseltamivir chemoprophylaxis before illness; and 7 of 8 were <59 months of age. Oseltamivir-resistance tests were not performed for these 8 patients because they all received oseltamivir therapy and their clinical symptoms resolved. Therefore, the possibility of transmitted resistance from contact patients was not demonstrated in this study.

All 11 patients were initially given the usual dose of oseltamivir (75 mg 2×/day in adults). After detection of oseltamivir resistance, treatment regimens were as follows: 3 patients were given high-dose oseltamivir (150 mg 2×/day in adults), 3 patients were given combination therapy (oseltamivir and amantadine; oseltamivir and peramivir; and oseltamivir, amantadine, and ribavirin, respectively); 3 patients were given zanamivir nasally; and 2 patients continued to receive oseltamivir.

Seven of the 11 patients had complications during treatment: 6 had viral or secondary bacterial pneumonia and 1 had acute respiratory distress syndrome (Table). Three patients died of pandemic (H1N1) 2009. Patient 3 died 15 days after confirmation of infection with pandemic (H1N1) 2009 virus and 10 days after the emergence of oseltamivir-resistant virus. Patient 4 died 15 days after confirmation of infection and 4 days after emergence of oseltamivir-resistant virus. Patient 8, who was infected with virus that had H275Y and I117M mutations, died 18 days after confirmation of infection and 4 days after the emergence of oseltamivir-resistant virus.

## Conclusions

Our nationwide surveillance of drug-resistant pandemic (H1N1) 2009 in South Korea indicated that most patients were children (<59 months of age) or immunocompromised. All isolates had the H275Y mutation in the NA protein, and 1 isolate also had the I117M mutation in the same protein.

Our finding that oseltamivir resistance developed in immunocompromised patients is consistent with those of recent case reports that described development of oseltamivir resistance in immunosuppressed patients receiving this drug (*3**,**4*). A recent study in Australia reported that 4 of 32 adult oncology and hematology patients were infected with oseltamivir-resistant virus with the H275Y mutation (*5*). In 3 of our patients (patients 3, 5, and 11) drug-resistant isolates appeared <5 days after initiation of oseltamivir therapy. Thus, we suggest that physicians be alert to emergence of oseltamivir-resistant pandemic (H1N1) 2009, particularly if there is treatment failure with oseltamivir or prolonged viral shedding is evident.

More than half of our patients were <59 months of age, indicating that a younger age may be a risk factor for infection with drug-resistant pandemic (H1N1) 2009. Clinical trials have reported oseltamivir resistance in <5.5% of children with seasonal influenza (*6*). Explanations for the higher rate of drug resistance in children than in adults are that children have a more protracted course of influenza, longer viral shedding times, and higher viral titers (*7*). Another explanation might be that we cannot rule out suboptimal dosing of oseltmaivir in children, although World Health Organization dose standards were used (*8*) and all pharmacies were given instructions on emergency compounding of oseltmaivir.

Development of resistance appeared to be caused by sporadic mutations. We found no evidence of transmission. Availability of pretreatment and posttreatment samples indicated that resistance to oseltamivir developed during treatment in >3 patients. One case report (*9*), 1 report of a cluster of cases in Vietnam (*10*), and 1 outbreak in a hematologic ward (*11*) documented patient-to-patient transmission of oseltamivir-resistant virus. One of our patients was infected with virus that had a novel NA mutation (I117M). A previous study indicated that the I117V mutation in avian influenza virus (H5N1) was associated with low-level oseltamivir resistance (*12*). The I117M mutation of NA may contribute to oseltamivir resistance, but it is not clear whether isolates with these 2 NA mutations have higher levels of resistance or virulence than the H275Y NA mutant.

We found few patients with drug-resistant pandemic (H1N1) 2009 in South Korea. However, our study was based on the nationwide surveillance system for drug resistance among patients with suspected oseltamivir treatment failures, which is different from other surveillance systems, in which all isolated viruses are tested. Thus, we cannot report the prevalence of drug-resistant pandemic (H1N1) 2009 and risk factors for drug-resistant virus infection in South Korea. Further studies are needed to monitor oseltamivir resistance, especially in immunosuppressed or pediatric patients when treatment failure with oseltamivir or prolonged viral shedding occur. Additional studies are also needed to determine proper oseltamivir dosing for children.

## References

[1] Cao B, Li XW, Mao Y, Wang J, Lu HZ, Chen YS, Clinical features of the initial cases of 2009 pandemic influenza A (H1N1) virus infection in China. N Engl J Med. 2009;361:2507–17. 10.1056/NEJMoa0906612 20007555

[2] Yi H, Lee JY, Hong EH, Kim MS, Kwon D, Choi JH, Oseltamivir-resistant infection of pandemic (H1N1) 2009 influenza virus in South Korea. Emerg Infect Dis. 2010;16:1938–42. 21122225 10.3201/eid1612.100600 PMC3294558

[3] Memoli MJ, Hrabal RJ, Hassantoufighi A, Eichelberger MC, Taubenberger JK. Rapid selection of oseltamivir- and peramivir-resistant pandemic H1N1 virus during therapy in 2 immunocompromised hosts. Clin Infect Dis. 2010;50:1252–5. 10.1086/651605 20345239 PMC2946636

[4] Chan PA, Connell NT, Gabonay AM, Westley B, Larkin JM, Larosa SP, Oseltamivir-resistant 2009–2010 pandemic influenza A (H1N1) in an immunocompromised patient. Clin Microbiol Infect. 2010;16:1576–8. 10.1111/j.1469-0691.2010.03212.x 20218988

[5] Tramontana AR, George B, Hurt AC, Doyle JS, Langan K, Reid AB, Oseltamivir resistance in adult oncology and hematology patients infected with pandemic (H1N1) 2009 virus, Australia. Emerg Infect Dis. 2010;16:1068–75. 10.3201/eid1607.091691 20587176 PMC3321901

[6] Whitley RJ, Hayden FG, Reisinger KS, Young N, Dutkowski R, Ipe D, Oral oseltamivir treatment of influenza in children. Pediatr Infect Dis J. 2001;20:127–33. 10.1097/00006454-200102000-00002 11224828

[7] Aoki FY, Boivin G, Roberts N. Influenza virus susceptibility and resistance to oseltamivir. Antivir Ther. 2007;12:603–16. 17944268

[8] World Health Organization. Updated interim recommendations for the use of antiviral medications in the treatment and prevention of influenza for the 2009–2010 seasons [cited 2010 Dec 23]. http://www.cdc.gov/h1n1flu/recommendations.htm

[9] Mandelboim M, Hindiyeh M, Meningher T, Mendelson E. Possible transmission of pandemic (HIN1) 2009 virus with oseltamivir resistance. Emerg Infect Dis. 2010;16:873–4. 20409390 10.3201/eid1605.091835 PMC2954011

[10] Le QM, Wertheim HF, Tran ND, van Doorn HR, Nguyen TH, Horby P. A community cluster of oseltamivir-resistant cases of 2009 H1N1 influenza. N Engl J Med. 2010;362:86–7. 10.1056/NEJMc0910448 20007549

[11] Moore C, Galiano M, Lackenby A, Abdelrahman T, Barnes R, Evans MR, Evidence of person-to-person transmission of oseltamivir-resistant pandemic influenza A(H1N1) 2009 virus in a hematologic unit. J Infect Dis. 2011;203:18–24. 10.1093/infdis/jiq007 21148492 PMC3086444

[12] Ilyushina NA, Seiler JP, Rehg JE, Webster RG, Govorkova EA. Effect of neuraminidase inhibitor-resistant mutations on pathogenicity of clade 2.2 A/Turkey/15/06 (H5N1) influenza virus in ferrets. PLoS Pathog. 2010;6:e1000933. 10.1371/journal.ppat.1000933 20523902 PMC2877746

